# Global Features of Gene Expression on the Proteome and Transcriptome Levels in *S. coelicolor* during Germination

**DOI:** 10.1371/journal.pone.0072842

**Published:** 2013-09-09

**Authors:** Eva Strakova, Jan Bobek, Alice Zikova, Jiri Vohradsky

**Affiliations:** 1 Laboratory of Bioinformatics, Institute of Microbiology, Academy of Sciences of the Czech Republic, Prague, Czech Republic; 2 Institute of Immunology and Microbiology, First Faculty of Medicine, Charles University in Prague, Prague, Czech Republic; University of South Florida College of Medicine, United States of America

## Abstract

Streptomycetes have been studied mostly as producers of secondary metabolites, while the transition from dormant spores to an exponentially growing culture has largely been ignored. Here, we focus on a comparative analysis of fluorescently and radioactively labeled proteome and microarray acquired transcriptome expressed during the germination of *Streptomyces coelicolor.* The time-dynamics is considered, starting from dormant spores through 5.5 hours of growth with 13 time points. Time series of the gene expressions were analyzed using correlation, principal components analysis and an analysis of coding genes utilization. Principal component analysis was used to identify principal kinetic trends in gene expression and the corresponding genes driving *S. coelicolor* germination. In contrast with the correlation analysis, global trends in the gene/protein expression reflected by the first principal components showed that the prominent patterns in both the protein and the mRNA domains are surprisingly well correlated. Analysis of the number of expressed genes identified functional groups activated during different time intervals of the germination.

## Introduction

Bacterial cell dormancy is a calmness stage of living cells that is characterized by minimal metabolic activity. In several bacteria, the beginning of the dormancy is accompanied by a transition into a morphologically and physiologically distinct form, which is known as a dormant spore. The spore formation ensures that the cell will survive under unfavorable conditions. The transition process is called sporulation and has been well studied, not only in the species of *Bacillus* and *Clostridium* but also in *Streptomyces*. *Streptomyces* are Gram-positive bacteria that undergo a complex cell cycle that involves morphologically distinguishable developmental stages. The stages include unicellular spores that develop to branching substrate mycelium, which gives rise to the apically growing sporangium known as aerial mycelium, from which the spores are formed. Although spore germination is usually accepted as the first stage of the cell cycle, from the one-cell point of view, it represents the middle part of the life of a single cell, which starts during aerial mycelium formation and finishes in the developed substrate mycelium. The reverse process, awakening the cell back into a metabolically active form, is called germination and, as opposed to the sporulation, is much less understood in *Streptomyces*. In this multicellular bacterium, the dormant spores are the only haploid unicellular state. The spores are surrounded by a coat that protects the cellular content from an environmental challenge and enables survival. The spores also possess intracellular nutrient and energy sources, such as trehalose [1] or most likely polyphosphates (volutin) [2]. From the sporulation phase, dormant spores are also accommodated with a protein apparatus, such as chaperones and cell wall hydrolases, which are effective during metabolism renewal [3]. It has previously been suggested that dormant spores are devoid of stable functional mRNA [4]. However, further experiments, using rifampicin as a transcription initiation inhibitor, have revealed that dormant spores possess a pool of mRNAs, which provide templates for early protein synthesis [5]. All of these sources that arise from sporulation are thought to ease cell survival by triggering energy metabolism, which starts when the dormancy is broken and lasts until the cells are adapted to exploit external nutrient supplies.

In a favorable milieu, spores lose hydrophobicity, which allows water influx, which, in turn, initiates germination. Cells re-activate their metabolism and develop into vegetative forms, building branching hyphae. Although several chemical or physical factors have been described for inducing germination, the molecular machinery that triggers the process remains unknown. Germination implies massive proteome reconstitution. This step is achieved by the utilization of spore compounds that are degraded during catabolic processes. Aggregated spore proteins that are preserved from dormancy are hydrated and re-activated during germination [6]. Cell wall hydrolases, such as RpfA and SwlA, provide the lysis of spore peptidoglycan to allow the entrance of external nutrients [7]. The re-activated chaperones GroEL, Trigger factor and DnaK were detected as assisting the reactivation of the proteosynthetic apparatus, which is fully accelerated during the first initial steps [8]. A systematic proteomic study that was recently conducted on streptomycete spore germination [3] revealed that the first newly expressed proteins are members of proteosynthetic machinery, proteins that are involved in differentiation, protein modifiers and other chaperones. Further protein expression evokes cellular responses to stress conditions and lasts approximately 1 hour. After this period, several protein regulators appear to take control over energy metabolism and further development. At this stage, the cell can respond to environmental conditions by direct gene expression.

Subsequent development to vegetative forms is a sequential process that is associated with the first DNA replication and an enhancement in the rate of RNA and protein synthesis [9]. Microscopically distinguishable germination tubes rise from the inner wall of spores and progress through the outer wall. The not-yet-fully defined end of the germination process is represented by the further protraction of the tubes, with the emergence of proteins that assist with cytoskeleton formation, such as DivIVA, FilP and FtsZ [3].

The correlation of the protein and mRNA expression levels has been most comprehensively reviewed by Abreu et al. [10], who summarize the knowledge of large-scale measurements on the level of whole transcriptomes and proteomes. Using 22 datasets that range from *E. coli* to Human, the authors document the correlation between protein and mRNA abundances, showing that a correlation coefficient (Pearson) ranges from 0.36 for *D. melanogaster* to 0.74 for *S. cerevisiae*. The authors also mention the importance of the quality of the measured data, which differs substantially between the protein and the mRNA measurements and is much lower for the proteomic data, especially in terms of the number of identified proteins in comparison with the mRNAs. For an exact comparison of the transcriptome and proteome expression, improvements in the proteome quantification are essential. A comparison of the time series of the gene expression of both types of data has much less representation in the literature than comparisons of one-point measurements. In streptomycetes, only two papers have addressed this issue, with the first comparing transcriptomic and proteomic time series measured during exponential growth of *S*. *coelicolor* [11] and the second using the same species but focusing on the late exponential and stationary phases [12]. Both of these papers used a similar information-extraction method, which was singular value decomposition (SVD) [11] or principal components analysis (PCA) [12]. Several other papers on this topic, mostly concerning yeast, were published over the past decade [13], [14], [15], [16], [17]. Although an absolute correlation between the protein and the mRNA was on the same scale as reported for other species (r = 0.63) [12], a striking similarity was found between the first PC loadings (eigenvectors), which were well correlated for the highest loadings, thus indicating a similarity in the expression between the protein and the mRNA of the backbone processes [13].

In this paper, we focus on the simultaneous analysis of gene and protein expression in *S. coelicolor* during the first 5.5 hours of germination, starting from dormant spores and sampled in 30 minute intervals. The obtained time series were compared using PCA and a correlation of the PC loadings with a time series of genes of the metabolic and regulatory functional groups.

The utilization of coding genes, i.e., how many genes and in what amounts they are expressed at individual time points, was performed on a symbolic level using generalized canonical law [18], [19]. Here, we focus on identifying the absolute numbers of expressed genes as a function of growth, an approach that allowed us to trace how different functional groups of genes are expressed in the course of germination.

## Materials and Methods

### 1.1. Spore cultivation

*S. coelicolor* A3 (2) M145 spores were pre-germinated in 2X YT media for 24 h (160 rpm, 30°C) [20]. Three milliliters of inoculum was transferred to solid agar plates (0.4% yeast extract, 1% malt extract, 0.4% glucose, 2.5% bacterial agar, pH 7.2) overlaid with cellophane discs and was cultivated for 14 days at 28°C. The harvested spores were used for germination in liquid AM medium. To boost the synchrony within the population, the spores were subjected to a 10 minutes heat shock treatment at 50°C. The protein and mRNA samples were collected at 30 min intervals starting from dormant spores until 5.5 hours of growth, obtaining samples at 13 time points. The phenotypic change occurring during germination is illustrated in the electron microscopy images of *S. coelicolor* spores (Figure S1. A, B) for the dormant spores (T Dorm, Figure S1. A) and for the germinating spores, 5,5 hours after germination initiation with grown germ tubes (Figure S1. B).

### 1.2. Proteomics

Details concerning the sample preparation, 2D electrophoresis, radio labeling, fluorescent staining and MS identification of protein spots can be found in our previous publication [3]. Here, we mention only the steps that are essential for this paper.

Germinating spores were radiolabeled with ^35^S Cysteine-methionine in 30 min radioactive pulses, except for the first time point (T0), which was labeled for 10 min during heat shock. Isolated protein samples were resolved by 2D gel electrophoresis using 24 cm strips with a pH range of 4–7. The second dimension was run on 12.5% polyacrylamide gels that were 25.5×20.5 cm in size, covering a Mw range of approximately 15–110 kDa. The gels were stained overnight with Sypro Ruby fluorescent dye and scanned on BioRad Phosphoimager FX for fluorescence intensity. Dried gels were exposed for 4 vdays to BAS cassettes (Fujifilm) and the protein radioactivity was determined using BioRad Phosphoimager FX. The stained and radioactive gel images were processed and compared using the software PDQuest 8.0.1 (Bio-Rad) to detect changes in the intensities for specific gel spots (proteins) over time and across replicates. Altogether, 54 2DE gels for Sypro Ruby staining and 50 radiolabeled 2DE gels were analyzed. Sypro Ruby-stained gels and radiolabeled gels were arranged into individual matchsets. The Sypro Ruby matchset reference gel contained 671 individual protein spots, and the radiolabeled matchset reference gel contained 404 spots. All of the gels were assembled into a single high-level matchset whose reference gel contained a total of 782 protein spots. All of the visible spots were picked from a preparative gel and were analyzed by mass spectrometry. Details about MS identification and a complete list of characteristics of MS spectra are given in the supplementary materials of our preceding paper [3]. The experiment was designed to cover both the experimental and biological variance, combining the measurements from different technical and biological replicates at one time point. The numbers of 2DE gels that were used for the replicates in different time points are given in Table 1.

**Table 1:** The number of 2DE gel images and microarrays that correspond to biological replicates analyzed for individual time points of the experiment.

Time [hours]	Number of Sypro Ruby-stained images	Number of radiolabeled images	Number of microarrays
Dormant	4	0	3
0	4	4	3
0.5	4	4	3
1	4	4	2
1.5	3	3	3
2	4	4	3
2.5	3	3	2
3	5	5	3
3.5	5	5	3
4	5	5	3
4.5	5	5	3
5	4	4	3
5.5	4	4	3

#### 1.2.1. Proteomic data normalization

The 2D electrophoretic spot intensities in individual gels were standardized by dividing the spot intensities by the total protein concentration loaded on a gel. The multiplicative factor was calculated from the accumulative gels (Sypro Ruby staining). We assumed that the logarithm of the intensities on the accumulative gels was normally distributed, with the means distributed around a common mean. Therefore, the means of all of the spot distributions for all of the stained gels were averaged, and a multiplicative factor that adjusted all of the distributions to the same mean was computed for each gel. Because a single gel contained both the fluorescent and radioactive labeling, the multiplicative factors derived for the Sypro Ruby-stained gels were also used to normalize the radioactive-based images (for details see our previous work [3]). This method also enables correct normalization for the radiolabeled gels, for which only a few electrophoretic spots could be identified in the first time points. Using a cumulative radioactive signal for their normalization would lead to an inadequate amplification of the first time points in the electrophoretograms.

To assess the degree of variance in the quantification of the relative protein abundance levels, we calculated the coefficient of variation (CV) for replicates within the individual time points for both types of labeling. The values were computed from the normalized data. The mean CV for Sypro Ruby stained gels was 0.39, and it was 0.54 for radiolabeled gels. These values were distorted by a time dependence; the highest CV was observed for the earliest gels and decreased over time (CV_max_ = 0.45, CV_min_ = 0.27 for the stained gels; CV_max_ = 0.62, CV_min_ = 0.4 for the radiolabeled gels). These values were comparable with those previously reported in the literature for 2D gel electrophoresis experiments (20–40%) [21] and were higher for the radiolabeled gels. The higher CV for the radiolabeled gels was caused mainly by the gels of the first time points, i.e., when proteosynthesis starts. The degree of variation is high both for technical reasons, when only a small number of spots appeared on a gel, and for the inherent variation among biological replicates, which is known to be high in *Streptomyces* in general. We attempted to overcome this problem by increasing the number of gels and sample replicates, which is almost two-fold higher than in a usual proteomic experiment of this scale (Table 1).

### 1.3. Transcriptomics

#### 1.3.1. RNA isolation from spores

To break the cells, we used a FastPrep-24 machine (Biomedicals) in which the spores were mechanically disrupted in tubes containing zirconium sand, two 4 mm glass beads, and 500 µl of lysis buffer [22] (50 mM Tris-HCl pH 8, 500 mM LiCl, 50 mM EDTA pH 8, 5% SDS) and 8 µl of RNAse inhibitors (Biorad). The disruption was made in 6 rounds for 35 s, while the tubes were re-chilled between each round. The samples were centrifugated at 14000 g for 15 min at 4°C, and the supernatant was used to phenol-chloroform RNA extraction, which was repeated twice. The RNA precipitated overnight in ethanol and 3 M Sodium Acetate at −20°C. Finally, the RNA was re-suspended in 50 µl RNAse-free water and 0.5 µl RNAse inhibitors and was cleaned from possible DNA remains using the DNAse-Free kit (Ambion). The RNA was stored in water at −20°C.

#### 1.3.2. DNA microarrays and data processing

The number of analyzed microarrays that represent 3 (or 2) biological and/or technical replicates for each experimental time point are given in Table 1. RNA quality control and gene expression levels were determined by Oxford Gene Technology (Oxford, UK) on Agilent DNA microarrays, covering the entire *S. coelicolor* genome, using OGT's standard Bacterial RNA amplification Protocol for the two-channel essay.

The acquired data were linear LOWESS normalized and filtered for background and flag information (from Agilent documentation) in the GeneSpring software to obtain genes that were significantly expressed above the background and to avoid the side effects of possible cross-hybridizations. This step reduced the number of entities on a single array from 43888 to 25312, which represented the outcome for 7115 genes of the original 7825. The data discussed have been deposited in NCBI's Gene Expression Omnibus [23] and are accessible through GEO Series accession number GSE44415 (http://www.ncbi.nlm.nih.gov/geo/query/acc.cgi?acc= GSE44415).

#### 1.3.3. Array normalization

The experiment included 37 arrays from 13 distinct time points of *S. coelicolor* germination. The arrays shared a common reference in the red channel (Cy5, beta channel), which was a mixture of RNA samples from all of the examined time points; the sample signal was recorded in the Cy3-labeled channel (alpha channel). The distributions of the Log2Ratio values (Log2Ratio =  log2 (Sample (Cy3)/Reference (Cy5))) from each array were centered to ensure that the medians and the median absolute deviations of all array distributions were equal. The centering was performed by subtracting the Log2Ratio median value of the array from each Log2Ratio converted measurement on the array and dividing it by the median absolute deviation. To eliminate array outliers, we filtered out the 0.02 quantile of the least and most intensive Log2Ratio values. The normalized Log2Ratios were returned to the original scale by exponentiation (creating normalized Ratios).

The time series of the relative mRNA concentration was obtained by averaging the normalized Ratios across biological and technical replicates at specific time points and across all of the gene replicate spots that were presented on the array. Before averaging, the outliers among the gene replicates at one time point were filtered using the Q-test (for 3–9 inputs) and the Pierce test (for >10 inputs).

The filtering caused the result that, in a few of the profiles, there was no value for certain time points. Such zero values were examined to determine whether they were placed between two non-zero time points. If the neighboring time points were non-zero, the missing value was linearly interpolated (which was performed for approximately 100 profiles of the total 7115). After filtering, the log2Ratio values were exponentiated to obtain the signal in its original scale. These measurements were arranged for individual genes into time series that form a “gene expression profile”, a term that will be used throughout this paper.

We further filtered out genes whose overall expression during germination was too low to be considered. The idea was to filter out genes whose microarray signal could be a result of array errors that were above the simple technical criteria. Therefore, a median expression level of the expression profile for all time points and biological and microarray replicates were computed. The expression level was defined as a raw signal from an individual chip spot, from the channel that recorded the fluorescently labeled mRNA sample (alpha channel). The median represents an overall expression level of individual genes. A logarithm base 2 of the expression profile medians was computed. The distribution followed a roughly lognormal shape, which indicated that there was a gap approximately at the position of the first quartile (data not shown). Therefore, we filtered out all of the genes whose log2median expression level was below the first quartile value. The genes that exhibit a single peak in the profile that would be otherwise filtered out were identified individually and were added to the final set. The final set contained 5385 genes. Among the removed genes, those that prevailed were the genes that have an unknown function and are unclassified and those that are associated with a secondary metabolism and are not expected to be expressed during germination.

### 1.4. Data treatment common to both proteomic and transcriptomic experiments

#### 1.4.1. Standardization

In the PCA analysis, we considered the changes in the gene/protein expression patterns rather than in the absolute values. Therefore, all of the profiles from all experiments were normalized to have the same mean and variance by subtracting the mean of the individual profile from each member point of the profile and dividing it by the standard deviation of the profile.

#### 1.4.2. Correlation

The Pearson correlation coefficient calculated below was tested under the assumption that each p-value is the probability of obtaining a correlation that is as large as the observed value by random chance, when the true correlation is zero. The p-value was computed using a t-statistic. If the p-value was ≤0.05, then the test was considered to be significant.

#### 1.4.3. Chi-square test

Chi-square statistic was used to compare the number of gene products in different functional groups in any of the selected sets (any of the sets that were selected by any of the analyses described below) in comparison with the abundance of the functional groups in the whole set (either proteomic or transcriptomic), as given in Table 2. If the p-value was ≤0.05, the test was considered to be significant.

**Table 2:** Functional classification of the genes of the S. coelicolor genome according to The Sanger Institute (ftp://ftp.sanger.ac.uk/pub/S_coelicolor/classwise.txt).

	Fluorescent labeling	Radio labeling	mRNA
Unknown function	46	26	1496
Chromosome replication	1	1	8
Chaperones	16	13	14
Protection responses	8	4	54
Transport/binding proteins	30	21	435
Adaptation	4	1	31
Cell division	2	2	14
Differentiation/sporulation	1	1	10
Macromolecule degradation	18	6	152
Macromolecule synthesis, modification	22	15	209
Amino acid biosynthesis	1	0	99
Biosynthesis of cofactors, carriers	6	6	88
Central intermediary metabolism	3	3	78
Degradation of small molecules	4	3	142
Energy metabolism, carbon	25	17	152
Fatty acid biosynthesis	1	0	45
Nucleotide biosynthesis	0	0	28
Secondary metabolism	0	0	163
Periplasmic/exported/lipoproteins	32	13	944
Ribosome constituents	5	4	60
Laterally acquired elements	0	0	76
Regulation/Two component system	3	3	121
Regulation/RNApolymerase core enzyme binding	4	4	68
Regulation/Defined families	3	2	325
Regulation/Protein kinases	0	0	35
Regulation/Others	5	4	175
Not classified (including putative assignments)	11	2	363
SUM	251	151	5385

## Results

### 2.1. Functional assignment of genes and proteins

The *S. coelicolor* functional genome database contains genomic information about 7825 genes that are assigned into three functional categories that have different levels of specificity. For our purposes, the most appropriate level was the second level, which classifies genes into 27 functional groups (Table 2). Table 2 lists the number of genes that are assigned to 27 functional classes for the microarray experiment and for the proteins that are both Sypro Ruby-stained and radiolabeled.

### 2.2. Principal components analysis (PCA) of proteomic and transcriptomic experiments

The correlation between mRNA and the protein abundances can be performed by comparing the values relative to a specific fixed time point that has a biological significance [12], and the following points are represented by the log base 2 ratio between the reference point value and the given point value. Such a point, in our case, represents dormant spores. In dormant spores, many of the proteins and mRNAs are not yet synthesized, and the first time point would thus often be represented by a zero value, which would cause the logarithm to approach infinity. Therefore, we could not make a direct comparison between the mRNA and protein abundances. We could only compare the shapes of the expression profiles that represent the kinetics of the expression of individual genes or proteins. All of the individual gene profiles in all of the three experiments were, therefore, normalized to have a zero mean and are used in further analysis (see paragraph 1.4.1). A correlation analysis between mRNA and protein kinetics showed higher correlation (Pearson correlation coefficient ≥0.95) only for 27.9% of profiles (Table S1), while the overall correlation through the dataset was rather low r = 0.05. Using the gene annotations and assigning each gene to a diverse functional group (Table 2), a functional analysis of the highly correlated profiles was performed. The functional analysis indicated that there is no bias among the highly correlated profiles toward a specific functional group.

**Figure 1:**
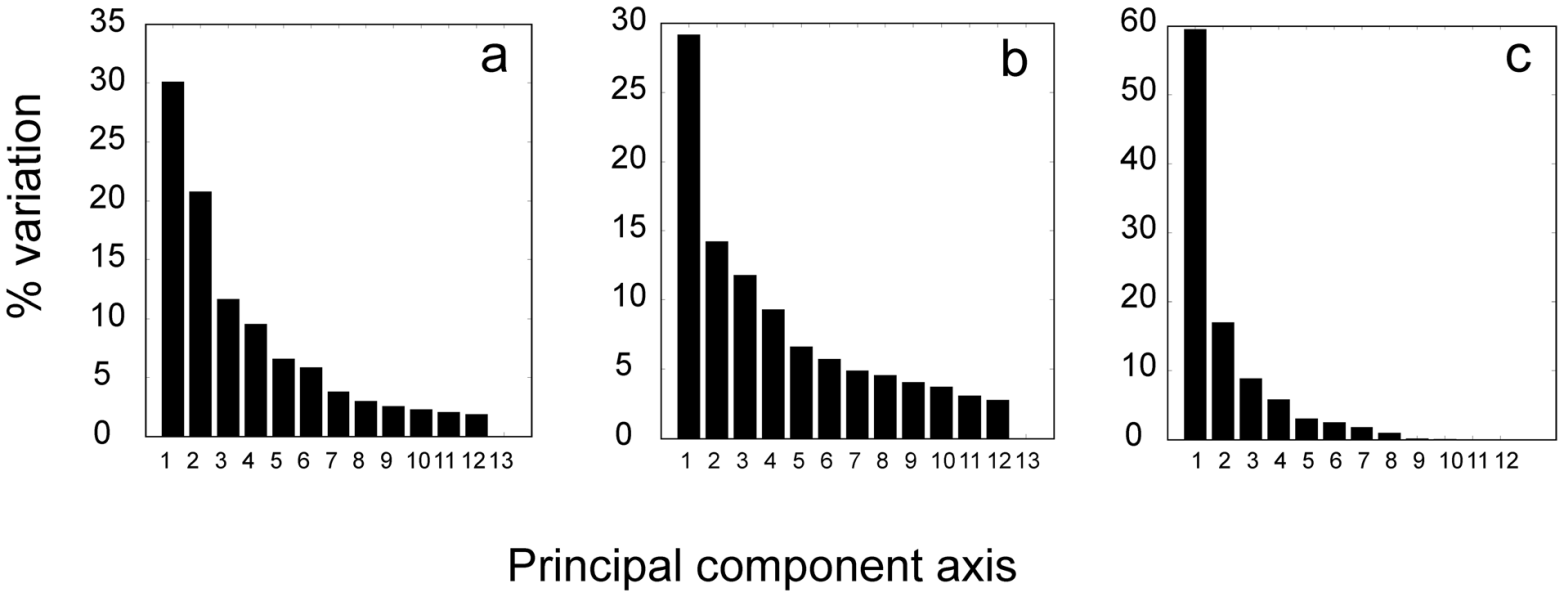
Percentage of variation (eigenvalues) accounted for by each of the 13 principal components. a – transcriptome, b – Sypro Ruby-stained proteome, c – radiolabeled proteome.

Therefore, instead of focusing on the correlation of individual kinetic profiles, it is more reliable to focus on extracting the common features of the system that are inherent in the expression time series. We chose principal components analysis, which allows identifying representative patterns of kinetic profiles according to their contributions to the overall variance of the dataset. The PCA was performed individually for the microarray experiment and the two proteomic experiments (the PCA or alternative SVD were also used previously in streptomycete gene expression studies [11], [12]). The first principal axis loadings (eigenvectors, PCs) bore 30% of the total variability for the microarray and Sypro Ruby-stained proteomic data and 59% for the radiolabeled proteomic data. The first three principal components represented 62%, 55% and 85% of the data variability of the three experiments (Figure 1).

**Figure 2:**
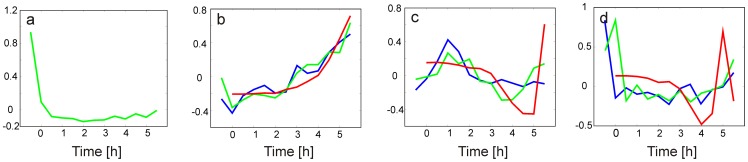
Profiles of the first PC loadings for the transcriptomic experiment and the two proteomic experiments. a) PC1 of the Sypro Ruby-stained proteomic experiment. b) blue – PC1 mRNA, green – PC2 Sypro, red – PC1 radiolabeling. c) blue – PC2 mRNA, green – PC3 Sypro, red –PC2 radiolabeling. d) blue – PC3 mRNA, green – PC4 Sypro, red – PC3 radiolabeling. The first time point in the radiolabeled profile is missing because this point represented dormant spores that could not be radiolabeled.

The first principal component loadings profile for the proteomic fluorescently stained experiment is shown in Figure 2a. Surprisingly, there was a striking similarity between the eigenvectors, found, when the eigenvector order of the proteomic fluorescently labeled experiment (PC (Sypro)) was shifted by one. Thus, a good correlation was found between PC1 (mRNA) and PC2 (Sypro), PC2 (mRNA) and PC3 (Sypro), and PC3 (mRNA) and PC4 (Sypro) (Figure 2). Examining the PC1 profile of the Sypro Ruby-stained proteome, a decline in the first two hours followed by a constant level is evident. We can, therefore, speculate that the PC1 (Sypro) is associated with the consumption of those proteins that were stored in the spores. In this case, such a phenomenon cannot be observed, neither on the mRNA nor on the radiolabeled protein levels. We would observe similarity among the eigenvectors only if the first PC (Sypro) is ignored; in other words, if the order of the PCs (Sypro) were shifted by one, then the result is the phenomenon that we indeed observe. Proteins with profiles that are correlated with the 1^st^ PC (Sypro) loading were distributed among the functional groups in the same way as the proteins of the whole set (data not shown). No bias toward a specific functional group was observed. Therefore, in further PCA analysis, we compared the transcriptomic and Sypro Ruby-stained proteome using this “shifted” order for the Sypro Ruby-stained proteome.

A comparison of the first principal component loadings for the three experiments is shown in Figure 2. Figure 2 shows good agreement between the profiles of the eigenvectors of fluorescently labeled proteomic and microarray experiments, documenting common trends in the gene/protein expression kinetics. The radiolabeled proteomic experiment followed the common trend only on the first eigenvector, and higher eigenvectors differed substantially. This difference can be explained by the different nature of the observed data, as the microarrays and Sypro Ruby-stained proteins represent protein or mRNA accumulation, while the pulse radiolabeled proteins represent the rate of protein synthesis. Because the accumulated protein expression level is the result of protein synthesis and degradation, while radiolabeling quantifies only the synthesis, their kinetics should, in principle, differ. The eigenvector profiles confirm that the accumulation kinetics (the result of balance between the synthesis and degradation), which is represented by the first eigenvectors, had a common trend, while the proteosynthesis rate followed different kinetics.

### 2.3. Gene expression profiles correlated with principal component loadings

Loadings or eigenvectors computed using singular value decomposition, a procedure similar to principal component analysis, that were computed from gene expression profiles, were shown to bear principal information about the kinetics of the processes associated with their shape [11], [13], [14], [15], [16]. They allow for the extraction of specific factors from the principal patterns of gene expression within a data matrix through comparisons with independent biological data and are the functional assignments of genes and their products. An analysis of the metabolic networks suggests the existence of different levels of cell control [24] (e.g., most of the metabolic flux in *E. coli* is controlled by only a small number of processes [24]). Such processes form the biochemical backbone for the physiological development; these are the processes that are associated with the programmed development of the cell. On this basic level, other regulatory processes controlling specific metabolic and regulatory activities at specific moments are superimposed. The whole scheme can be depicted as a hierarchy of processes at different levels of specificity. The final kinetics of gene expression is thus determined by the weighted superposition of all of these contributions. If these processes are uncorrelated, then the PCA can deconvolute the information in the profiles of the whole transcriptome or proteome and identify the principal kinetic shapes. Correlation of these shapes, i.e. PC loadings, with individual gene expression profiles and functional annotations of the genes of correlated profiles can reveal the hierarchy of the processes that control the developmental phase under analysis, with the first principal components bearing the most important physiological processes. Deconvolution technically means sorting the PC loadings and their profiles according to their information content level, which is given by their eigenvalues. The first few loadings (usually 3), thus bear most of the information about the kinetics of the underlying processes. The expression profiles of the genes that are correlated with the first principal component loadings thus represent the processes that have the highest importance for the developmental program that they record. Identifying the genes and their functional assignments, whose profiles are correlated with the first few PC loadings, can identify the metabolic and regulatory pathways that are fundamental for the studied developmental processes. In the following section, we focus on the correlation analysis of gene expression profiles with the first three principal components, i.e., PC1-PC3.

Because the numbers of proteins in the individual functional groups were rather low, in the analysis of the correlation of expression profiles with PC loadings (given in the next paragraphs), we analyzed only the transcriptomic time series.

#### 2.3.1. PC1

The first principal component shows after drop down in the first time point a continuous increase in the gene product accumulation for all three types of data (Figure 2b). PC1 for mRNA (PC2 for sypro Ruby-stained proteins) increased its accumulation throughout the whole germination period. PC1 for radiolabeled proteins kept increasing almost exponentially. Because all of the expression profiles were normalized to have the same mean, we cannot obtain an absolute value for the expression of individual genes that is correlated with this profile. However, we can presume that the gene products that had the PC1 shape were either already accumulated in the dormant spores or were synthesized immediately after the initiation of germination, and their accumulation kept growing during the 5.5 hours of germination.

The Pearson correlation between the PC1 and the gene expression profiles identified 1403 genes (26% of the total) that were significantly correlated (p<0.05). A statistical test that compared the abundance of the gene functional groups in the correlated set and the whole set of potentially expressed genes showed that the PC1-correlated set contained over-represented genes for the functional groups “Cell division” (the relative abundance in this set was 2.4× higher than in the full set), “Macromolecule synthesis, modification” (1.6×), “Metabolism of small molecules” (1.73×), “Energy metabolism, carbon” (1.5×), “Ribosome constituents” (2.2×), and “Protein kinases” (2.07×).

The enrichment in the functional groups “Cell division”, “Macromolecule synthesis, modification” and “Ribosome constituents” suggests that the processes represented by PC1 are characterized by the initiation of the spore basal metabolism after breaking the dormancy (as is the translation machinery) and launching the active management with available energy sources (the over-represented “Energy metabolism” group). The list of PC1-correlated genes is given in Table S2.

#### 2.3.2. PC2

The second principal component loading profile was characterized by a peak at approximately time point 4 (1 h) (Figure 2c). Altogether, 791 gene expression profiles were correlated with PC2. Over-represented in this group were the genes that were assigned to groups that are associated with the synthesis of macromolecules, which are either associated with their modification (“Macromolecule synthesis, modification”, which was 1.7 times more present in the PC2-correlated set than in the full set of genes) or with providing building material for protein biosynthesis (the functional group ”Amino acid biosynthesis” (2×)). Other regulatory proteins that were not associated with the main regulatory groups (i.e., two component systems and transcription), which are mostly annotated as DNA binding proteins (functional group “Regulation/Other”), were 1.8× over-represented. Additionally, genes of the group “Transport/binding” were significantly over-represented, as were genes of the group “Regulation/Defined families”. Genes that belonged to the “Regulation” group were mostly transcriptional regulators of the R families (such as LysR, TetR, MerR or MarR). Not surprisingly, the genes of the groups “Differentiation/sporulation”, “Cell division”, and “Laterally acquired elements” that are associated with the processes different than germination, were totally absent.

Unlike the genes that correlated with PC1 (which represent basal metabolism), the over-represented groups associated with PC2 (a peak at 1 h) are those that reflect the actual developmental state of the cell and direct the further growth of the cell. In this group, we found an over-represented diverse spectrum of genes that have regulatory functions from the groups “Regulation/Defined families”, “Regulation/Others” and “Transport/binding”. This result indicates that, by means of the regulation factors whose expression peaks are at 1 h, the cells can detect signals from and respond to the environmental conditions. This finding is in agreement with our previous proteomic analysis [3], in which, at the time point preceding the peak at 1 h, we detected the synthesis of most of the regulatory and transport/binding proteins in the germinating spores. The subsequent decrease after the peak at 1 h in the PC2 profile is most likely a result of the synthesizing, transport and regulatory functions of the protein members of the PC2 groups that lead to the controlled expression of the other components that build the growing cell. The list of PC2-correlated genes is given in Table S3.

#### 2.3.3. PC3

The third principal component loading profile was characterized by an initial maximum at the beginning of the germination and an increase at the end of the measured period (5.5 hours, Figure 2d). A total of 342 genes were found to follow this profile. An analysis of the over-represented functional groups showed more abundant genes in only two groups: “Amino acid biosynthesis” (2.54×) and “Regulation/Protein kinases” (4×). All of the other groups had the same relative presence of the genes as the relative presence that was found in the whole set. The list of PC3-correlated genes is given in Table S4. The “Amino acid biosynthesis” group was also enriched among the genes that correlated with PC2, and the “Regulation/Protein kinases” group was over-represented in the genes that correlated with PC1. In contrast to PC1 (increasing), the character of the PC3 kinetic trend (an abrupt decrease in the first hour) suggests that a rapid switch occurred within the first hour and was mainly in the expression of the genes from the two over-represented groups. The switch in the usage of the amino acid biosynthesis group in the first hour can be explained as a reaction of the metabolism to a sudden supply of amino acids from the AM medium that was used for cultivation, when the cell was adjusting its metabolism to the current environmental conditions. Metabolic changes might also be associated with the requirement of a different specific set of protein kinases compared with the set that is needed just after the germination activation.

Principal component analysis of the gene expression data showed that the most important aspects are the processes that are associated with the first three principal components. Unlike in our previous work [11], where we found a strong association between the fifth PC and antibiotic production, the higher principal components could not be associated with any developmental process (data not shown). The number of genes that were associated with the principal components decreased rapidly with the decreasing eigenvalues of the corresponding principal components. A total of 1403 gene expression profiles were correlated with PC1, 791 were correlated with PC2 and 342 were correlated with PC3. The experiment was also designed to cover the biological variability that occurred when each repeat from each time point measurement was collected from different biological replicates (see the Methods section). Although it was not statistically proven, the biological variability was the main source of the experimental error, and the lowest PCs were apparently associated with the experimental noise. Our analysis proposed that the PC1-associated processes are connected with launching the basal metabolism (a more detailed analysis of the basic metabolic processes supporting this statement is given in the following paragraph). Comparison of PC1 profile with the course of DNA synthesis (Figure S2), where approximately at 2.5 hours of growths DNA replication starts, shows moderate correlation with PC1 for microarray and proteomic Sypro experiments, indicating association between PC1 correlated genes/proteins and first DNA replication. Making a definite statement about the correlation between PC1 associated genes and DNA synthesis is complicated by rather high variance of both proteomic and transcriptomic experiments reflected in the PC1 profile. Although it cannot be undoubtedly confirmed such observation has to be mentioned.

The PC2-correlated processes represent the response of the cell to the actual environmental and/or inner conditions through corresponding regulatory pathways. PC3 reflected processes that are suppressed after germination initiation as a reaction to the medium composition detected by the cell. Genes that are associated with these processes were identified and are available in the supplementary materials.

### 2.4. PCA and major functional groups

In the preceding paragraphs, genes in the major functional groups were examined for their correlations with the first principal eigenvectors (PC1). In the next section, we focus on genes that are essential for re-activating metabolism after breaking dormancy, i.e., the genes of energy metabolism, nucleic acids and protein synthesis, and their association with the principal kinetic shapes represented by first principal eigenvectors. In addition, the genes of the stress response were examined because germination can be considered a reaction to the stress that is associated with the rehydration of the originally dry spores.

#### 2.4.1. Energy metabolism

**Figure 3:**
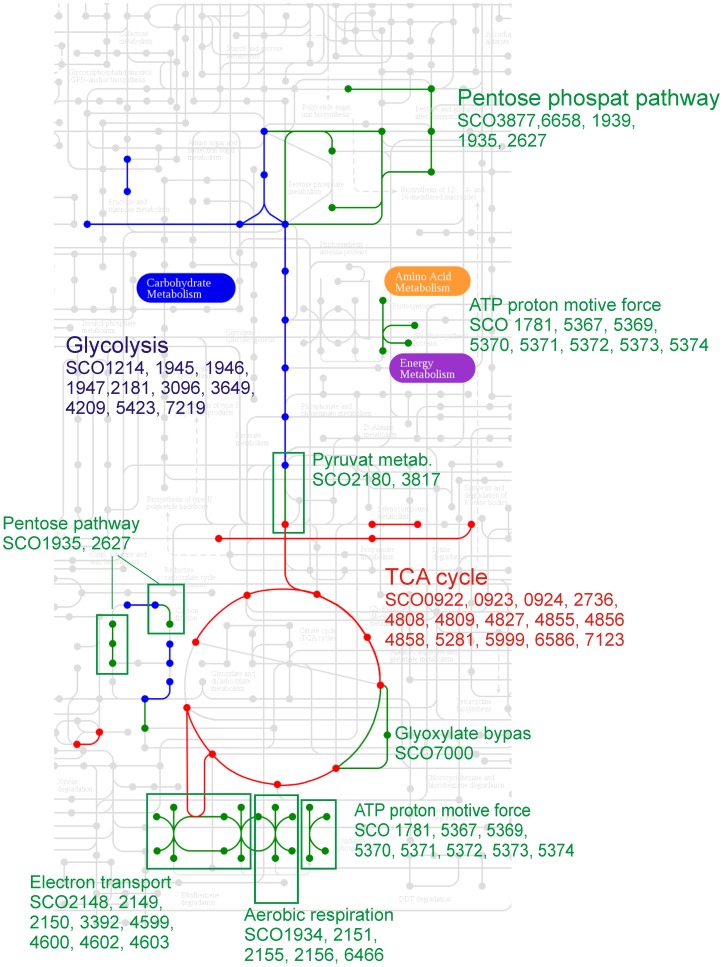
A visualization of genes significantly correlated with PC1, which are involved in primary energy metabolism. The basis of the illustration was made in KEGG mapping tool.

We use the specific gene annotation and a pathway mapping tool given in the KEGG database (Kyoto Encyclopedia of Genes and Genomes, http://www.genome.jp/kegg-bin/show_organism?menu_type=pathway_maps&org=sco) to investigated mRNA expression profiles involved in the primary energy metabolism. The most of the energy pathways-associated genes are significantly correlated with PC1 (Figure 3), including genes comprehended in TCA cycle, pentose phosphate pathway and glycolysis. Figure 3 shows mapping of PC1 correlatad gene expression profiles on to the KEGG pathway map.

For the genes annotated in KEGG, the significant correlation with PC1 was found for 38% of TCA cycle genes, including the subunits of succinyl CoA synthase (SCO4809,6586), citrate synthase (SCO2736), malate dehydrogenase (SCO4824) and acetyl transferase (SCO7123), for 22% of glycolysis genes and 25% of genes from pentose phosphate pathway.

A significant correlation with PC1 was also found for mRNAs that encode for the pathway that leads from the glyceraldehydes 3-phosphate to pyruvate (*gap* (SCO1947), *pgk* (SCO1946), and *eno* (SCO3096)) and for the oxidative phosphorylation genes *cox1* (SCO2155,2156) and *qcrB* (SCO2148) and ATP synthase (SCO5374). For none of the genes from TCA cycle, pentose phosphate pathway and glycolysis a correlation with PC2 was found.

#### 2.4.2. Stress response

The systematic annotation of stress response genes is not available; thus, we extracted the names of the stress response genes from the two most relevant resources, i.e., GenBank and StreptoDB (http://strepdb.streptomyces.org.uk). Altogether, 46 genes that represent heat, cold and starvation shock were analyzed. Approximately half of them were correlated with PC1. Heat shock proteins did not show any correlation with PC1. In contrast, 6 of the 8 cold shock proteins that were found were positively significantly correlated with PC1 (SCO4684, 527, 4505, 5921, 3748, and 3731). The general stress protein 50S ribosomal protein L25 *ctc* (SCO3124) had the highest correlation with PC1 (r = 0.89) among all of the stress proteins. Catalase *catA/C* (SCO0379,0560) showed a negative correlation with PC1. Starvation genes (*pstB* (SCO4139), *pstS* (SCO4142), *regX3* (SCO4230), a phosphate transporter (SCO4228), alkaline phosphatase (SCO2286, 1906, 3790, 5140) were mostly negatively correlated or not correlated with PC1. Only *regX3* (SCO4230) and dehydrogenase (SCO2490), a general stress protein, were found to be significantly correlated with PC2. Other stress proteins did not show any significant correlation with PC2.

#### 2.4.3. Nucleic acids and protein synthesis

Proteosynthetic genes were highly correlated with PC1. Of 53 proteosynthetic genes (groups “Amino acyl tRNA synthase tRNA modification” and “Proteins – translation and modification”), 80% were significantly correlated; the correlated genes primarily comprised tRNA synthase genes and the elongation factors Ts (SCO5625), Tu (SCO4662), P (SCO1491) and G (SCO4661) and the translation initiation factors IF-1/2 (SCO 4725, 5706). In contrast, RNA synthesis and DNA replication genes were correlated only moderately (30% of 113 were significantly positively correlated). Among the positively correlated genes, the subunits of DNA polymerase III (SCO1827, 2003, 6084, 3541) or helicase (SCO 2952, 1167) were found.

The correlation with PC2 was always lower than that of the PC1 genes and was highest for the genes that were involved in the “DNA replication repair” group (26% of 80).

The analysis of the association of specific functional and metabolic groups with the first two eigenvectors showed that PC2 did not correlate with almost any of the selected groups and genes, while PC1 was correlated with specific functional groups. As PC1 represents principal kinetic shape controlling germination, genes correlated with PC1 represent principal pathways controlling germination. The principal expression profile (Figure 2b) shows that the genes having this profile are relatively highly expressed in dormant spores, their expression drops just after germination initiation and after that, their relative expression continuously increases until the end of measured period. Expression profiles having this shape belonged mostly to the genes of energy metabolism that are involved in basic metabolic processes, such as the TCA cycle and its associated pathways Genes that were highly correlated with PC1 were involved in nucleic acids and protein synthesis, including the main translation factors. In agreement with the results of the global analysis of the association of general functional groups with eigenvectors (paragraph 2.3), the basic processes of germination, with the kinetics represented by PC1, involve biochemical and regulatory pathways that are indispensable in accelerating the primary energy metabolism to an increased level and are required for the cell to become competent to develop into vegetative forms. These data also indicate that, as a response to the increased demand of the new proteome constitution and, therefore, the capacity of a proteosynthetic apparatus, the sole re-activation of aggregated proteosynthetic components is insufficient and must be accompanied by the *de novo* synthesis of its members.

From the group of stress responses, the genes associated with cold shock were highly correlated with PC1. This result confirms the hypothesis suggested by Strakova et al. [3], i.e., that germination involves gene expression processes that resemble cell responses to stress conditions.

### 2.5. Absolute gene/protein expression levels

It was shown above that the genes associated with PC1 bore principal expression profiles associated with genes driving germination. The remaining question was, whether the PC1 associated genes had different levels of expression in comparison with the whole mRNA dataset.

The samples on the chips in a transcriptomic experiment were labeled and organized such that the samples were in one channel (the alpha channel, labeled with one fluorescent dye) and the standard (a mixture of samples from all time points, see Methods) were in the other channel (the beta channel, with a second fluorescent dye). The time series in all of the above analyses used the ratio between the alpha and beta channels. This arrangement decreases the measurement variance but does not allow for a direct comparison of the absolute expression levels among the different genes because the standard hybridizes differently with different mRNA probes that are immobilized on the chip. Because the mRNA sample repeats were randomly distributed among the different arrays and equal amounts of mRNA were always loaded on the chip, we were able to use the alpha channel alone and compare the absolute expression levels of the individual genes. The absence of the standard increases the variance of the averaged expression values of individual mRNA levels but should not influence the overall trend; in other words, the expression profiles obtained both from the alpha channel only and from the normalized data should be correlated. An analysis of the correlation coefficient distribution between the alpha channel and normalized ratios showed that 75.6% of the genes were significantly correlated (p< = 0.05), with a maximum at r = 0.87. We also found that the lowest correlation was associated with profiles that had an overall low expression level in the profile.

**Figure 4:**
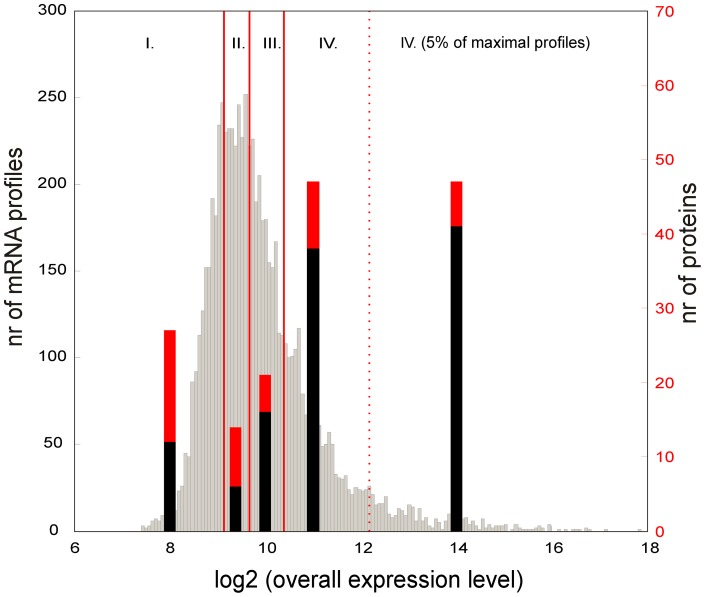
Distribution of the medians of the gene expression profiles, given in absolute units, as measured in the microarray alpha channel. The distribution is divided into 4 quartiles (roman numbers) and the last 5% of the most highly expressed genes. Vertical bars represent the number of proteins of Sypro Ruby-stained (red bars) or radiolabeled (black bars), in the proteome that corresponds to the genes in the given quartile.

For the individual genes from the alpha channel (representing the absolute gene expression level) the log-base2 distribution of the medians of the expression time series is shown in Figure 4. This distribution, even in the logarithmic scale, was heavily tailed toward highly expressed genes. When compared with the number of identified proteins (the thick bars in Figure 4) that correspond to the genes in a given quartiles of the distribution, it is apparent that most identified proteins were within the fourth quartile of the distribution, especially in the most highly expressed 5% of the genes. The relationship between the expression level of mRNA and the number of corresponding proteins that are expressed is apparent.

When examining the functional characteristics of the genes in the most highly expressed 5% percent, the genes were significantly enriched in the groups “Regulation/RNApolymerase core enzymes binding protein” (2.4×), “Ribosomal constituents” (which correspond to 80% of all of the ribosomal genes predicted in the *S. coelicolor* genome), and “Chaperones” (60% of all of the genes predicted in the genome). The possible role of chaperones in germination was discussed in the work of Bobek et al. [8]. There were also significantly more genes that were involved in the translational machinery (the elongation factors, RNA polymerase subunits), the energy metabolism and, strikingly, the cold shock genes.

Comparing the analysis of the association of genes with the first principal components (paragraph 2.3), a similarity between the highly expressed group and the group of genes that are correlated with the PC1can be observed. The similarity implies that the principal regulatory groups, which are necessary for germination control and progression, have not only the kinetic profile defined by the PC1 shape but also belong to the overall highly expressed group. The presence of cold shock genes in both groups is interesting. As mentioned in [25] bacterial cold shock proteins sequences are conserved among species, however their role can differ in various organisms and furthermore not all of them are induced by cold shock. As the genome annotation is frequently made by homology search, the annotation of the genes as cold shock genes may be caused by the way the genes were annotated. We have checked all the cold shock genes mentioned above for their appearance in the literature, but we didn't find any particular reference dealing with their function in *Streptomyces*. It is apparent that these genes are essential for the progression of germination, but their actual role remains a puzzle, and it will require further work to determine their function in germination.

Detailed inspection focusing on individual genes shows that the group of most expressed genes comprised important transcription regulators: sigma factors, anti-sigma factors and anti-anti sigma factors. Interestingly, we detected a very large expression of the gene for the alternative principal sigma factor HrdD (SCO3202), whose function has not yet been revealed in *S. coelicolor*. The group also contained both genes for SigH anti-sigma factor Prs (SCO5244) and the gene for its partner switch, an anti-sigma factor antagonist BldG (SCO3549), which were recently shown to interact directly and participate in switching-like activation/deactivation of the sigma factor SigH [26]. Similarly, the regulation based on a switch-like mechanism was proposed for anti-anti-sigma factor ArsI (SCO3067), the SigI anti-sigma factor antagonist [27]. In addition to the gene for an extracytoplasmic function (ECF) sigma factor SigE (SCO3356), which regulates genes that are involved in cell wall biosynthesis [28] among the maximally expressed genes of regulators, *sigD* (SCO4769) and genes predicted to encode sigma factors SCO0038 and SCO4908 (which products have still unspecified function) were found. Finally, the detected members of this enhanced regulatory group were the genes for co-expressed partners, sigma factor SigR (SCO5216) and its anti-sigma factor RsrA (SCO5217), which together create a control system that is sensitive to changes in the intracellular redox balance [29]. Interestingly, in the enriched functional group “Biosynthesis of cofactors and carriers” (2×), we found several genes that were previously reported as SigR target genes [30], [31], such as thioredoxin enzymes (SCO0885, SCO1084, SCO3889), which assist in reducing disulfide bonds that are unnatural for the intracellular environment. Additionally, genes that were involved in the translation machinery were found in the highest expressed gene group, including the genes for elongation factors P, G, Tu, Ts (SCO1491, 4661, 4662,5625) and initiation factors IF-1 and 2 (SCO4725, 5706). As mentioned above, among the highly expressed genes, the cold shock genes (SCO527, 3731, 4295, 4505, 4684, 5921) were found.

### 2.6. Analysis of coding gene utilizations

Further, the changes in number of highly expressed genes during germination within individual functional groups were investigated.

**Figure 5:**
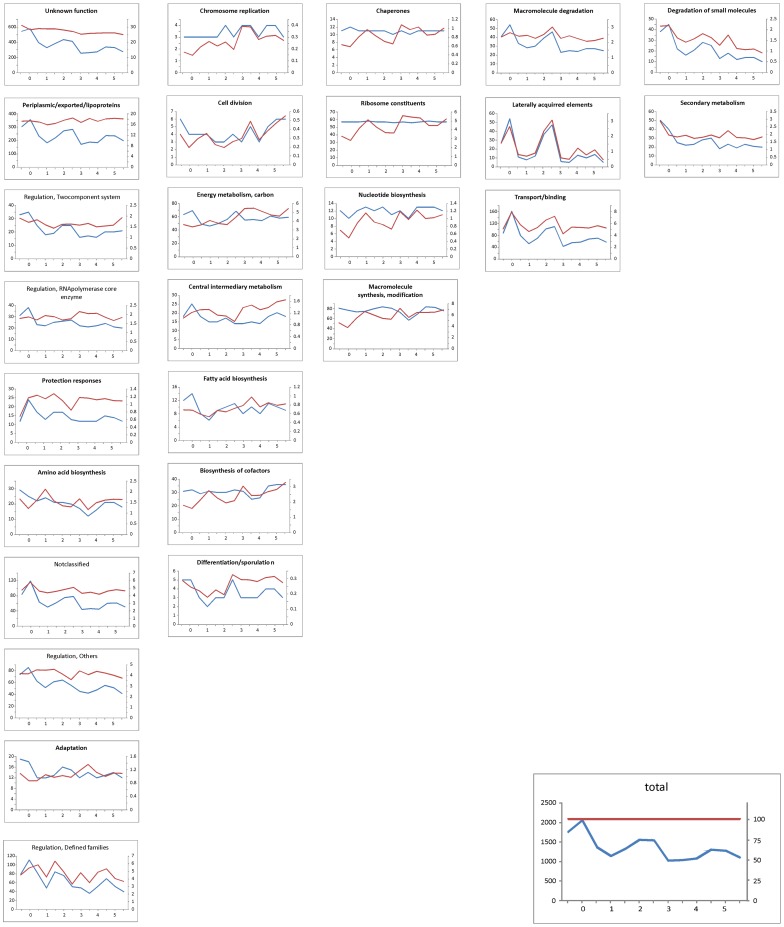
The number of expressed genes of different functional groups over the course of germination. Blue curve (left vertical axis) – absolute numbers of expressed genes at a given time point, red curve (right vertical axis) – number of expressed genes relative to all of the expressed genes, in terms of the percent. Horizontal axis – time [hours]. Individual functional groups are ordered in columns according to the similarity of the profiles.

We used data from the alpha channel, as described in the previous paragraph. To select genes that were highly expressed and to eliminate the influence of the high variance of the low-expressed genes on the analysis, we chose the threshold level of the third quartile of the distribution calculated from alpha channel signals. All of the mRNAs that had a signal higher than this threshold were selected for further analysis. Then, for each time point, all of the genes with an expression value above this threshold were identified and sorted into 27 functional groups (as defined in Table 2). The numbers of highly expressed genes assigned into each individual functional group were counted at each of the 13 time points, generating a time series of the number of highly expressed genes in the individual functional groups (Figure 5, the blue curve). To validate whether the threshold value (defining high expression genes) could influence the profile shapes of the numbers of expressed genes, the same process was repeated with a threshold values that were equal to the first and second quartile (data not shown). The results showed that the selection of the threshold value has no effect on the shapes of the profiles given in Figure 5, and therefore, we used the original threshold value of the third quartile.

The resulting profiles are shown in Figure 5. Figure 5 shows that the overall number of expressed genes during the course of germination exhibited two peaks (Figure 5, caption “Total”); the first occurred in T0, and second occurred after approximately 2.5 hours of growth (time points 6–7). After the second peak, the expression stabilized at a level of approximately 1200 highly expressed genes. It is striking that there is a relatively high number of mRNAs that are found in dormant spores (ca. 1600). A previous study of *S. granaticolor* in experiments using the transcription inhibitor rifamycin revealed that dormant spores preserve pre-existing mRNAs, which are expressed at the beginning of germination [5]. In our previous work on the proteomic dataset [3], we found that several newly synthesized proteins appear just minutes after germination initiation. The finding that a relatively large number of mRNAs exist already in spores could suggest that these proteins were synthesized from this stock.

To compare the trends in individual functional groups with the overall trend, the individual profiles were divided by the general pattern and were multiplied by 100 (red curves in the graphs of Figure 5). Such curves show a deviation from the general pattern (caption “Total” in Figure 5) for the given functional group. The group of “Regulation/Protein kinases” was excluded because it contained only a few mRNAs (0–3). Additionally, the groups of “Cell division” and “Differentiation/sporulation” contained a small number of mRNAs (5 on average), but they were retained for comparison.

The time series of the abundance of the genes of different functional groups can be dissected into 5 principal patterns according to the development of the relative number of genes (red curve in Figure 5) that were expressed during germination. The first and largest group of functional assignments copied the profile of the general pattern (blue curve and flat red curve in first column of graphs in Figure 5). It can be expected that the genes in the “Unknown function” and “Not classified” groups will follow the general pattern because they contain an uncharacterized mixture of genes. Aside from the above-mentioned functional groups, this group contained mainly regulatory genes and genes involved in the synthesis of macromolecules and amino acids.

The second largest group showed an increase in the relative number of expressed genes over time (red curves in second column of graphs in Figure 5), including the genes of “Energy metabolism”, “Central intermediary metabolism” and “Fatty acid biosynthesis”.

The third group was formed by the genes that were expressed always in the same numbers, regardless of the development phase (blue curves in third column of graphs in Figure 5). Not surprisingly, this group was formed by “Ribosome constituents”, which are expected to be constitutively expressed, and “Chaperones”. This group also contained the genes of “Nucleotide biosynthesis” and “Macromolecular synthesis”, which similarly followed a constant trend.

Groups of “Macromolecular degradation”, “Laterally acquired elements” and “Transport/binding” followed the general trend but had a much higher emphasis.

The genes of “Secondary metabolism” were surprisingly expressed in non-negligible numbers (approximately 30 (12.5%) of all secondary metabolism genes), and its numbers declined over time and had a maximum in the dormant spores. These transcripts usually originate from the sporulation stage, in which the antibiotics are produced, and they are subsequently degraded. The presence of several members of antibiotic gene clusters might also suggest that their enzymatic activity is required in germination. However, none of those proteins were detected here. A detailed inspection of the individual genes did not reveal any specific and/or continuous gene clusters for the synthesis of secondary metabolites. Because the group of “Secondary metabolism”, as defined by the Sanger Institute, also includes a number of other genes that are not directly associated with biosynthetic clusters (such as lipoproteins), an over-representation of this group is most likely not associated with secondary metabolite production but is instead associated with those genes that are not directly involved in the synthesis of secondary metabolites.

Similar pattern could also be observed for the genes of the group “Degradation of small molecules”.

## Discussion

A correlation analysis between proteomic and transcriptomic data showed a rather low correlation between the mRNA and the accumulated protein expression profiles. Of the 247 genes/proteins that were investigated, 27.9% were highly correlated (a correlation interval of ≥0.95). This finding is consistent with other analyses on *Streptomyces* species, which also found a correlation for approximately one-third of the genes that were expressed during the stationary phase [10], [11], [12]. Several similar studies, which were mostly performed in yeast [32], [33], [34], [35], have reported varying, but still rather low, correlations between mRNA and protein abundances, which range from 0.21 to 0.74 (Pearson correlation). The variability between datasets mostly goes to the account of the translation and posttranslational processes and also the errors in the measurements, which are quite high.

Whenever a comparison between proteomic and transcriptomic data is performed, two sets that substantially differ in size are compared. While proteomic experiments, which are made either by 2D electrophoresis or mass spectrometry, can quantify usually hundreds of proteins, microarrays provide information about thousand of genes. The sets differ in size by one order of magnitude, which leads to a comparison of biased selection of only highly abundant proteins with unbiased microarray expression data. We must admit that, except for the analyzed intersect, we do not and will not know what is the “true” correlation (across the whole proteome and transcriptome), until the proteomics will be able to quantify a more representative part of the real proteome. Therefore, instead of focusing on the correlation of individual expression profiles, it is more reliable to focus on extracting the common features of the system that are inherent in the gene expression time series. One of these methods is the principal components analysis, which, when used to analyze a set of gene expression profiles, allows us to identify patterns of gene expression that contribute to the pathways controlling the observed process. Comparing these patterns for different experiments (proteomic or microarray) could say how much these methods identify common features of the system and could show how much they give similar results. The analyses made so far on *Streptomyces* [11], [12] show very good agreement between proteomic and transcriptomic temporal data at the level of the first eigenvectors, which confirms that the fundamental processes are controlled in a coordinated fashion on both the transcriptomic and proteomic levels. The pulse-labeled data differed substantially, which is most likely caused by the different nature of the measured values – the accumulation of the mRNA and protein fluorescence staining vs. the expression rate for the pulse radiolabeling. By correlating individual gene expression profiles with the first eigenvectors, we were able to identify the metabolic and regulatory pathways that control the fundamental processes during the germination of *S. coelicolor*.

The analysis of highly expressed genes showed a correlation between the expression levels of the mRNAs and the corresponding proteins; genes that are highly expressed on the mRNA level are also highly expressed on the protein level. Functional analysis of most of the highly expressed genes showed that the highly expressed genes are also those that are correlated with PC1. This comparison shows that the principal regulatory groups that are necessary for the germination control and progression are, overall, highly expressed and have a kinetic profile that is defined by the shape of the first principal component.

Respecting the limitations of the proteomics approach, we utilized a more detailed analysis that addresses individual genes and functional groups, and we focused on mRNA data and interpreted the gene expression in the sense of genome utilization over the course of the experiment. The analysis of the number of genes of individual functional groups that were expressed during the course of germination showed that a relatively high number of mRNAs existed already in the dormant spores. The mRNA synthesis peaked at the first time point of the germination and declined until the end of the observed period, with a small local maximum at 2.5 hours. This result, which is in agreement with previous proteomic analysis [3], suggests that dormant spores already contain most of the genetic material that is necessary for the spore germination initiation that was stored, most likely in aggregates, that stabilize both the mRNA and proteins and which, after rehydration, can readily initiate the growth of the spores. The peak of the macromolecule synthesis that was found at the first 30 min after initiation supports this statement.

In interpreting both the transcriptomics and proteomics data, we could not go below a certain level of generality given by the inherent information content that is different for both of the data sources. However, analyzing large-scale gene expression data using statistical methods such as PCA can give us insights into how biochemical and regulatory processes work in the cell on the systems level.

## Supporting Information

Figure S1 **The phenotypic change occurring during germination is illustrated in the electron microscopy images of** ***S. coelicolor*** **spores at primary magnification of 30 000 times.** A) Dormant spores (T Dorm). B) Germinating spores, 5,5 hours after germination initiation with grown germ tubes.
(TIF)

Figure S2 **Comparison of the principal components with DNA synthesis.** To observe the status of DNA replication during germination the cells were germinated in 50 ml AMK media with radioactive (5.5 µCi) nucleobase ^14^C Thymine. Radioactive thymine incorporated into newly synthesized DNA molecules within replication. We collected 2×100 µl of cell suspension in 20 min intervals up to 5 hours of germination. The samples were washed to remove unincorporated radioisotope and remaining radioactive signal of the cells was measured. The average radioisotope signal CPM (count per minute) of two samples at each examined time point is represented by the black cross. The black dash line indicates that the first DNA replication occurred around 2.5 h after germination initiation where the significant increase of radioisotope signal was detected. Up to 140 min the radioisotope signal is constant (approximately 110 CPM) and represent background but from 160 min the sudden increase (roughly doubled to 230 CPM) correspond to the doubling of DNA in the first DNA replication.
(TIF)

Table S1 **Genes with highly correlated scores (Pearson correlation > = 0.95) of Sypro Ruby stained proteins (PC2) and mRNA (PC1).**
(XLSX)

Table S2 **Genes that have an expression profile that is correlated with PC1.**
(XLSX)

Table S3 **Genes that have an expression profile that is correlated with PC2.**
(XLSX)

Table S4 **Genes that have an expression profile that is correlated with PC3.**
(XLSX)
