# Supplementary figures and images for: Global Features of Gene Expression on the Proteome and Transcriptome Levels in S. coelicolor during Germination
Source: PLoS One. 2013 Sep 9;8(9):e72842. doi: 10.1371/journal.pone.0072842 (PMC3767685; doi:10.1371/journal.pone.0072842)

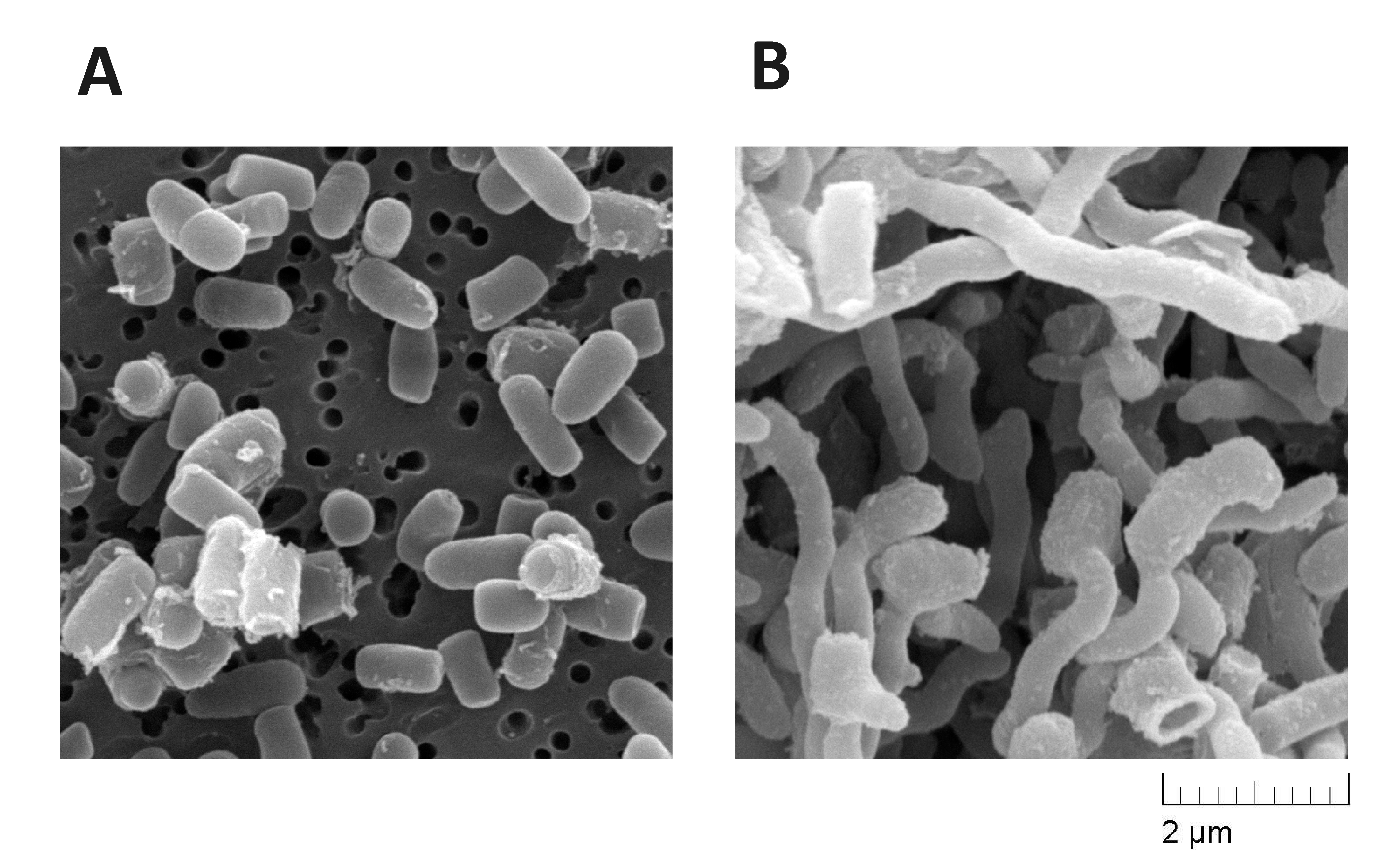

Supplement: Figure S1 — The phenotypic change occurring during germination is illustrated in the electron microscopy images of S. coelicolor spores at primary magnification of 30 000 times. A) Dormant spores (T Dorm). B) Germinating spores, 5,5 hours after germination initiation with grown germ tubes. (TIF) [file pone.0072842.s001.tif]

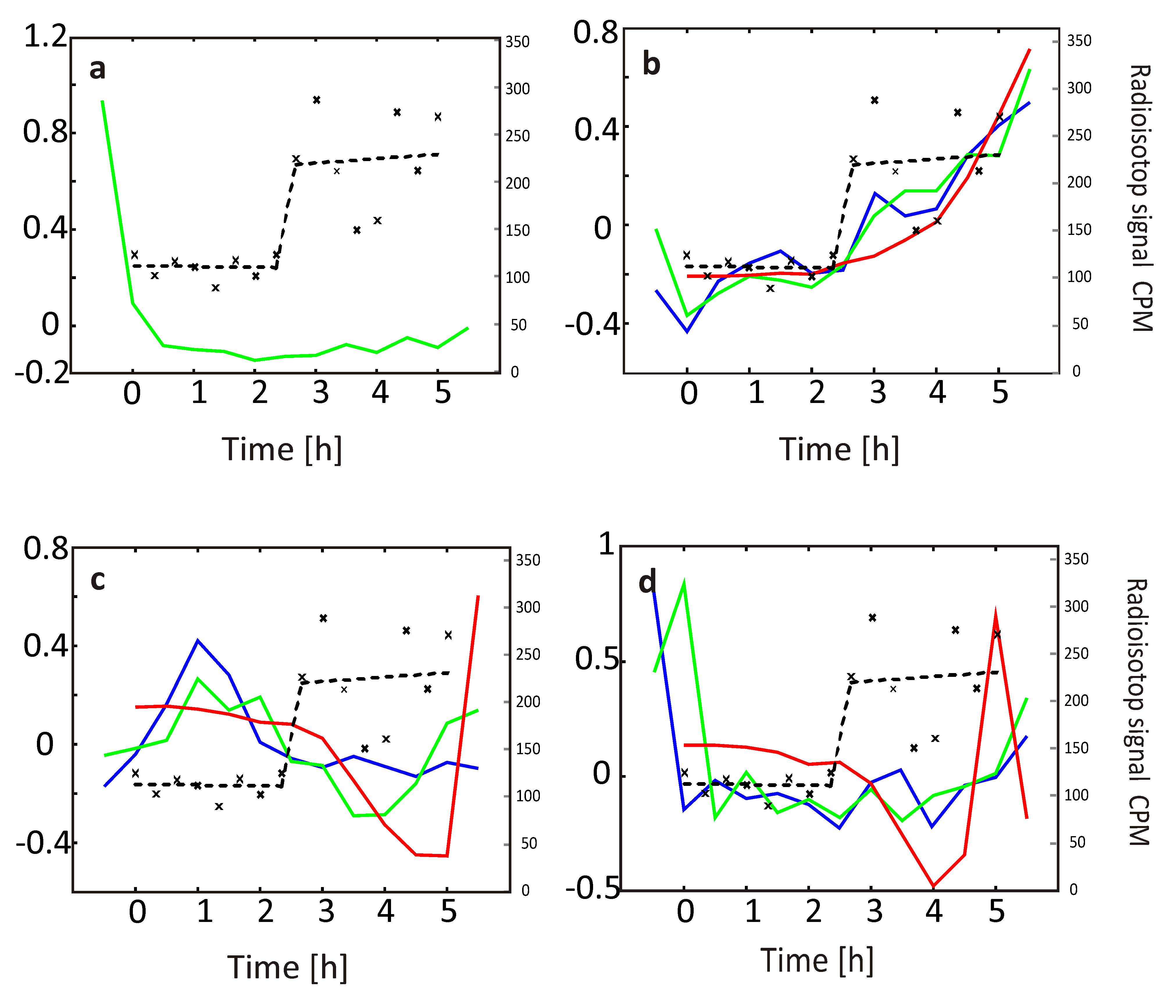

Supplement: Figure S2 — Comparison of the principal components with DNA synthesis. To observe the status of DNA replication during germination the cells were germinated in 50 ml AMK media with radioactive (5.5 µCi) nucleobase 14C Thymine. Radioactive thymine incorporated into newly synthesized DNA molecules within replication. We collected 2×100 µl of cell suspension in 20 min intervals up to 5 hours of germination. The samples were washed to remove unincorporated radioisotope and remaining radioactive signal of the cells was measured. The average radioisotope signal CPM (count per minute) of two samples at each examined time point is represented by the black cross. The black dash line indicates that the first DNA replication occurred around 2.5 h after germination initiation where the significant increase of radioisotope signal was detected. Up to 140 min the radioisotope signal is constant (approximately 110 CPM) and represent background but from 160 min the sudden increase (roughly doubled to 230 CPM) correspond to the doubling of DNA in the first DNA replication. (TIF) [file pone.0072842.s002.tif]
